# Leveraging deep learning for the detection of socially desirable tendencies in personnel selection: A proof-of-concept

**DOI:** 10.1371/journal.pone.0329205

**Published:** 2025-08-05

**Authors:** Tony C. Lee, Matthias Ziegler

**Affiliations:** Institute of Psychology, Humboldt Universität zu Berlin, Berlin, Germany; University of Delhi, INDIA

## Abstract

We propose a deep learning-based method for detecting Socially Desirable Responding (SDR)—the tendency for individuals to distort questionnaire responses to present themselves in a favorable light. Our objective is to showcase that such novel methods can be leveraged to design instruments that have the potential to measure this construct in an effective way. Participants’ tendency to engage in SDR was initially modelled by specifying a latent variable model from Big Five personality scores, using data from 91 participants in a job application simulation (Big Five questionnaire and video introduction). Nonverbal visual cues (5,460 data points following data augmentation) were extracted from the participants’ video presentations in form of sequences of images for training a transfer learning model designated as *Entrans*. The objective of *Entrans* is to discern patterns within these cues in order to detect whether sample participants manifest higher or lower SDR tendency. We conducted a regression-based prediction task to train and evaluate *Entrans*, resulting in a promising performance (MSE = .07, RMSE = .27, ρ = .27). A further analysis was conducted using a classification-based prediction task, which corroborated the potential of *Entrans* as a tool for detecting SDR (AUC = .71). These results were further analyzed by a Grad-CAM method to elucidate the underlying model behaviors. Findings suggest that the middle and lower parts of the face were the regions relied upon by *Entrans* to identify individuals with higher tendency of SDR in the classification task. These tentative interpretations give rise to the suggestion that socially desirable responding in a questionnaire and impression management in a job interview might share a common underlying cause. While the detection of SDR during personnel selection presents a significant challenge for organizations, our proof-of-concept demonstrates how machine learning might be leveraged to develop practical solutions as well as addressing theoretical questions.

## Introduction

The complexity of filling job vacancies is a challenge for organizations. Interviews are one of the most used tools for assessing candidates’ knowledge, skills, abilities, and personality [1]. In addition, recruiters tend to rely on personality questionnaires to increase the accuracy of their assessments or to pre-select candidates for a potential interview [2]. However, previous studies have shown that such questionnaires as well as interviews can be affected by a well-known drawback: *impression management*, a form of *Socially Desirable Responding* (hereafter SDR) [3,4]. Applicants’ SDR has been shown to distort the construct and criterion validity of both questionnaire and interview assessments [5–9].

Deception like SDR is extremely difficult to detect, especially in overt behavior like in an interview situation. DePaulo and her colleagues [10] meta-analyzed 158 ‘cues to deception’ reported in 116 primary studies. Their results revealed that these verbal, nonverbal, and other miscellaneous cues showed no discernible links, or only weak links, to deceit. In fact, humans are barely able to pick up lies on the basis of nonverbal cues, verbal cues, and even with the assistance of physiological and neuroscientific methods [11]. While psychologists in industrial-organizational fields have suggested that recruiters should review suspicious questionnaire results during the interview [12], studies have shown that this suggestion is difficult to implement in real-world settings [13]. Instruments such as social desirability scale and lying scales have proved to be of limited success [14]. Meanwhile, even though it has been evidenced that SDR affects interview and questionnaire results, it remains unclear whether the two phenomena reflect the same psychological processes. It is rarely discussed in the field of personality psychology whether the SDR behavior manifested in one assessment measure is related to the one in another measure. Understanding this phenomenon may be important in explaining why SDR detection is difficult, despite the efforts of I-O psychologists to develop tools to detect SDR, mostly in vain [14,15].

To fill the research gap and advance our knowledge on the cross-method nature of SDR, we experiment with a deep learning method to shed light on a possible association of SDR across questionnaires and overt behavior. Our research question investigates whether deep learning models can predict an individual's SDR tendencies in a questionnaire by identifying and leveraging nonverbal cues gathered from overt behavior. Thereby the current study provides the psychology community and practitioners with a novel approach to studying faking. We treat this research approach as a proof-of-concept that has the potential to help understand complex phenomena such as SDR. The current proof-of-concept attempts to demonstrate how deep learning methods might be used to not only develop practical applications (e.g., detecting SDR), but also advance theoretical knowledge (e.g., comparing different manifestations of a construct).

## Social desirability and personnel selection

There was a long-running debate about whether SDR was a phenomenon that only occurred in the laboratory or whether it was a real-life behavior that could compromise selection outcomes [16]. Today, most evidence supports that SDR does indeed occur in questionnaires [17] and interviews [3] during real-life recruiting processes. It is therefore important for organizations to tackle the issue of SDR when assessing their candidates.

With the help of factor analytic approaches, it is possible to model the variance due to SDR in a single factor. The interpretation of this factor as SDR has been corroborated using different experimental or statistical approaches [9,18,19]. This factor is also referred to as the “ideal employee factor” [20] because it captures the psychological processes that encompass the tendency for applicants to present themselves as the most suitable candidate, even to the extent of distorting their actual personality [13]. Although there is research suggesting that this factor does not affect test criterion correlations of personality domain scores [9], the variance does distort construct validity and reliability estimates [21,22]. This means that selection based solely on such questionnaire data may capture unintended outcomes. In addition, research shows that SDR affects who is hired [23], suggesting a detrimental effect of SDR on the integrity of the selection process. From a practical perspective, modeling SDR variance does not help solve the general problem of how to deal with SDR. At best, recruiters can correct questionnaire scores to obtain more trustworthy assessment results [24], but this method requires sufficient sample sizes. To date, there is no best solution as to how to handle SDR in questionnaire assessments using classical rating scale formats. For interviews, there appears a similar body of research underscoring the presence of variation due to impression management in self-presentation data (e.g., [3]).

Overall, previous studies show consensus on the existence of SDR in questionnaires and interviews, and this variance has an impact on the results of personnel selection. Although experts in the field have proposed several approaches to deal with this undesirable effect, the implementation of these approaches is not without problems. This also means that as long as organizations continue to use interviews and questionnaires as selection tools, it will be necessary to improve their ability to detect/accommodate SDR. Later, we will address the question whether the variance due to SDR in questionnaires is linked to the variance in interviews.

## Machine/Deep learning and SDR detection

The use of machine learning has become increasingly popular in psychological research in recent years [25,26]. As opposed to traditional statistical analysis, researchers tend to implement machine learning in an exploratory manner, casting a large number of independent variables (or “features” in machine learning terminology), expecting to identify a robust association with one or more dependent variables [27]. In this way, machine learning opens the door to new types of studies that have rarely been attempted.

Since machine learning is based on advanced statistical models commonly used in computer science, it offers researchers in fields such as psychology the opportunity to overcome analytical limitations. For example, shrinkage algorithms (e.g., Lasso, ridge, and elastic net) can alleviate collinearity limitations by imposing a penalty on large regression coefficients; the “support vector machine” algorithm can map feature-outcome relations in multidimensional space to optimize linear bounds between values of the dependent variable in a nonlinear operation; and simple ensemble models such as “random forest” use many iterations to create strong learners in each decision tree (multiple models trained from many smaller subsets of features and data sets) [28]. Deep learning, powered by (artificial) neural networks, can automatically learn features from the data, handle large-scale data where the number of input variables exceeds the number of subjects, and reduce the maximum prediction error and provide more robust results [29].

In settings such as job recruitment, Batrinca et al. [30] investigated the extent to which machine learning algorithms can predict applicants’ Big Five personality traits; Nguyen et al. [31] trained a deep learning model from nonverbal cues to assess applicants’ “hirability”; and Suen et al. [32] used deep learning to profile applicants’ personality in asynchronous video interviews, to just provide a few examples. Building on previous research on the relation between personality and job performance [33], organizational psychologists are increasingly interested in implementing machine learning techniques to enhance personality prediction and advance organizational development. However, some have cautioned that these algorithms should be operated in a theory-driven and replicable manner to reduce the risk of Type I errors [34] or to avoid spurious findings that are not generalizable [35]. Hickman et al. [36] found compelling evidence of the validity of machine learning models for personnel selection when trained on interviewer-reported questionnaires, as opposed to self-reported ones.

In the previous section, we outlined the challenges of assessing an individual's SDR tendency using conventional tools, whether in the form of questionnaires or interviews. A novel machine learning approach holds promise for changing the existing situation. While there is ample research on the use of machine learning to develop automated personality prediction models for selecting personnel, only a few studies have investigated the potential of this approach for automated SDR detection. Baumgartl et al. [37] employed a random forest algorithm to classify dichotomized SDR in their conference paper. In a more recent study, Röhner et al. [38] investigated the capability of machine learning classifiers, including logistic regression, random forest, and XGBoost, to identify SDR in a questionnaire and confirmed the potential of machine learning in this field. To broaden this area of inquiry, we offer a deep learning method for identifying SDR that builds on previous investigations relying on questionnaires and interviews. Our technique for developing a SDR detection model diverges from the methods employed in the two preceding studies in terms of extent, predictors, model architecture,performance evaluation, and model explainability. Our aim is to develop an intelligent system capable of predicting an individual’s SDR tendencies by analyzing nonverbal cues extracted from job applicants’ brief video self-presentations. Such an algorithm may alert recruiters to SDR intentions adopted by applicants. From a theoretical perspective, an effective model as such may help to identify a possible systematic relation between SDR in questionnaires and SDR in overt behavior, helping to bridge two rich research traditions.

## Method

### Data

In this study, we rely on the Self-Presentation Corpus collected by Batrinca et al. [30] to build a deep learning model for SDR detection. The provided dataset consists of video recordings of 91 participants, including 47 males and 44 females. Additionally, their self-rated Big Five personality (domain level) scores are also included in the dataset. The age of the participants ranged from 18 to 52 years, with an average of 29. Participants were recruited from the staff of a research center (n = 24), students (n = 43), and non-academic adults (n = 24).

The initial experiment (see [30]) simulated a recruitment process. As such, all individuals were requested to attend the site and record a brief self-presentation, ostensibly at the beginning of an interview, using the video communication tool Skype. A webcam situated atop a monitor recorded the participants’ responses. Prior to arriving at the laboratory, all participants were required to complete an online Big Five questionnaire. It was an Italian adaptation of the Big Five Inventory questionnaire [39], consisting of 50 items (ten per Big Five trait), in which participants were asked to rate each item on a 7-point scale (1 = not at all, 7 = very much). Afterward, a Skype call was initiated by the pretend “interviewer” to communicate remotely with the participants. At the start of the video recording, the interviewer posed the same question to each interviewee, asking them to introduce themselves without any preassigned instructions or topics. The duration of the interviewees’ self-introductions varied between 25–180 seconds. All participants recorded their videos in the same space with identical settings. It can be reasonably concluded that the video background should not serve as a confounding variable that would mislead the model.

### Estimating SDR

The concept that SDR affects items across scales and that this can be modeled as an additional factor has been discussed since the publication of the paper by Schmit and Ryan [20]. With the rise of confirmatory factor analyses, this additional factor was often modeled as a bifactor, explaining variance in all items in addition to the substantive factors (e.g., [19]). A variety of experimental methodologies have been conducted to substantiate the nature of this latent factor. For example, Bäckström and colleagues have created neutral items (i.e., neither socially desirable nor undesirable) and could show that such items do not contain a SDR bifactor [18,40,41]. In a different approach using an experimental design, Ziegler and Bühner [9] could show that the SDR bifactor can be attributed to conscious impression management. Other studies [42,43] have also used this approach further providing support for the notion of modeling SDR as a common factor. It could be argued that such a bifactor captures other response styles (e.g., acquiescence) or common method variance (see [44]). If this were the case, it would be highly improbable to identify any relation to the behavior exhibited in the video. Following this rationale, the present study can be considered as potentially providing further corroborating evidence for modeling SDR as a bifactor. Accordingly, we constructed a latent variable model using the R package *lavaan* [45] with regression method to extract a latent SDR factor from the Big Five domain scores of our collected data.

### Model training strategy

#### Model prediction task.

This study aims to train an advanced machine learning model capable of predicting an individual’s socially desirable tendencies in settings such as recruitment. We will administer two model prediction tasks: a regression prediction task and a binary classification task. These are supervised machine learning tasks designed to predict the unseen value of labels from a set of related features. The distinction between classification and regression lies in the approach that while the regression task requires a model to predict continuous values, the classification task requires the model to predict categorical classes. Moreover, these two predictive tasks rely on distinct metrics for assessing the model’s predictive performance. In a regression task, ML practitioners typically use metrics such as root mean square error (RMSE) or mean absolute error (MAE) to measure the deviation of their predictions from the actual values; in a classification task, practitioners commonly rely on metrics such as accuracy, AUC, precision, recall, or *F*_*1*_ score to measure how many correct cases a model successfully predicts.

As previously stated, the extracted SDR scores were designated as the criterion for the two prediction tasks. In the regression task, the continuous values of SDR were employed as the criterion, whereas in the classification task, the SDR scores were transformed into binary categorical values. Such an operation is a common practice in the field of machine learning, as evidenced by García et al. [46]. However, in psychology, dichotomization is often a source of concern. Cohen [47] already cautioned that artificially dichotomizing a variable, for instance by dividing a sample into high and low binary categories, results in a loss of information and tends to diminish the overall observed effect. Moreover, the classification of data near the split criterion is susceptible to measurement error, which can lead to erroneous classifications, particularly in the vicinity of the data split (see also [48]).

In accordance with the prevailing conventions in the field of psychology, we prioritize the results derived from regression-type prediction as the reference point for evaluating the performance of machine learning. Additionally, we conducted the classification task as a supplementary approach to demonstrate some useful aspects when transforming a model for categorical prediction. For example, the transformation allows for the application of sophisticated model interpretation techniques, particularly those designed for classification tasks tailored to computer vision.

While classification studies often rely on the mean or median as a cutoff for dichotomization, such an approach would likely overestimate the percentage of respondents who exercise SDR tactics. To mitigate this issue, we based our cutoff threshold on results from previous findings that estimated the percentage of respondents utilizing SDR tactics in selection contexts. For example, the study by Griffith et al. [17] concluded, based on quasi-experimental data, depending on the accepted margin of error 30–50% of applicants distorted their answers in a questionnaire. Zickar et al. [49] analyzed an applicant data set using mixed Rasch models and reported a proportion of up to 80% of respondents faking. We based our decision for a cutoff on this result and combined it with the goal to avoid a totally imbalanced dichotomization. Moreover, considering that the current data were derived in a laboratory setting, we chose to select a higher proportion of respondents using SDR which reflects findings suggesting stronger SDR effects in the lab, compared to real applicant settings (e.g., [17]).

Accordingly, for our classification task, we used a threshold of 75 percentiles to dichotomize the actual SDR scores into binary classes (higher and lower level of SDR). We are aware that this transformation is not without consequences: on the one hand, we have created artificial, somewhat naive, binary SDR categories at the cost of losing a certain amount of information from their original continuous values; on the other hand, our SDR categories are skewed and the number of values between the two categories is imbalanced: the total number of lower SDR values was three times larger (75%) than the total number of higher SDR values (25%). From a technical standpoint, this increases the difficulty of model learning, as a naive model is likely to fail to learn from the training data and will attempt to predict only the majority class (lower SDR). For this reason, we advise readers to consider our classification prediction as a supplement to the results of the regression task. It is worth noting that, in the regression task, we followed the recommended practice of normalizing continuous values between 0 and 1 in order to facilitate model computation.

#### Optimizing model performance.

In machine learning, the generalizability of a model often hinges on its robustness (see [50]); that is, its predictive accuracy on unseen data. It is commonly thought that training a model in supervised learning typically requires a considerable amount of well-annotated data to ensure robust performance, while data scarcity often leads to overfitting and poor model performance. However, obtaining a large amount of training data remains challenging in real-world application settings owing to time and resource constraints. Yet, determining the sample size needed to train a model a priori is often arbitrary in machine learning (see [51,52]). Moreover, recent research discourages the excessive pursuit of size in machine learning by demonstrating that a small sample size can be appropriate for a study, provided it is of good quality, as it is not affected by the winner’s curse and minor analytical manipulations. In some cases, increasing the sample size does not help improve the model robustness [53]. Some researchers (e.g., [54]) provide evidence that validating a machine learning model by resorting to an appropriate validation method (e.g., nested cross-validation) produces robust and unbiased performance estimates regardless of sample size.

In line with these perspectives, we have developed adequate approaches based on existing work, with the objective of adapting the training schemes for building an effective SDR detection model that overcomes the constraint of sample size. Our approaches include: 1) exploiting feature extraction techniques to optimize the input space; 2) employing data augmentation techniques to enhance model learning; and 3) designing fine-grained deep learning model architectures capable of identifying meaningful patterns for SDR detection. Our first approach addresses the challenge of sample size by extracting more representative information from the raw data. Numerous studies have demonstrated that incorporating pertinent input into the model greatly improves its performance (see [55]). Based on previous insights into a likely relation between self-presentation and nonverbal behavior [10,56], we will extract sequences of images from the video presentation of the sample participants that are expected to reflect nonverbal behavior. These images will serve as input data for fitting our model to discriminate participants’ SDR tendencies.

The second approach to building a robust SDR detector involves augmenting data complexity through artificial transformations applied to the extracted features. This method, known as data augmentation, is widely recognized as one of the most effective means of alleviating the limitations of sample size (see [57]). Basic image augmentation techniques, such as geometric alteration (e.g., image rotation) or non-geometric alteration (e.g., image flipping or cropping, noise injection), increase the complexity of the data, thus force the model to learn harder to pinpoint the “right” patterns, which should lead to the improvement of model performance and more generalizable predictions. For our case, we resort to three data augmentation techniques, namely dropout, affine transform and mix-up, to increase the complexity of the training dataset. To maintain the authenticity of the data during the model validation phase, we avoid applying data augmentation to the unseen data used for final validation.

The third approach aims to address concerns related to model performance by using sophisticated model architecture, such as multilayer neural networks (see [58,59]). Traditional machine learning models such as logistic regression, support vector machines, or random forests exhibit limitations, particularly regarding input feature type, model flexibility, and learning ability. In contrast, numerous deep learning models built on the architecture of neural networks are effective in identifying underlying patterns in various types of input data. Furthermore, deep learning models hold promise for improving the potency of the original model by being trained on top of a more sophisticated model. This process, known as transfer learning, involves the primary model receiving knowledge distillation from another model [60]. In essence, transfer learning is the process of fine-tuning a pre-trained model for a specific task using the knowledge gained from the original training. In the field of computer vision, ImageNet, a proficient deep learning model that has been trained to recognize images from millions of samples, serves as a common transfer learning base model for creating new fine-grained models [61]. Similarly, abundant real-time translation apps and chatbots grounded in natural language processing rely on the prevalent large language models, which has been trained on billions of text samples [62]. In a more metaphorical sense, the practice of transfer learning in model training is similar to schooling, whereby the new model can draw on the already accumulated intelligence to solve various technical problems related to model robustness and prediction generalization.

### Model training procedure

Using the aforementioned strategies, we have trained a transfer learning model to predict the socially desirable tendency of participants in our study. Previous literature has provided insight that people may deliberately regulate nonverbal behaviors for the purpose of deception during a self-presentation, but their attempt to control nonverbal behaviors may also induce traces of deception [56]. In light of these findings, we leveraged the key frame extraction technique – combining it with optical flow analysis [63] – to extract meaningful sequences of 60 image frames per participant video with a frame size of 512 × 384 × 3 (the height and width of the color image) during the feature engineering phase. The images were extracted on a frame-by-frame basis with subsequent aggregation. Our design choice was primarily driven by implementation considerations. In the field of computer vision, transforming image data for data augmentation is a widely used technique, supported by numerous advanced methods and packages. With well-documented tutorials, applying data augmentation to images is relatively straightforward. By supplying participants’ sequential images as informative input data, we expected to train a model capable of discriminating participants’ SDR tendency by means of their nonverbal behaviors.

**Fig 1:**
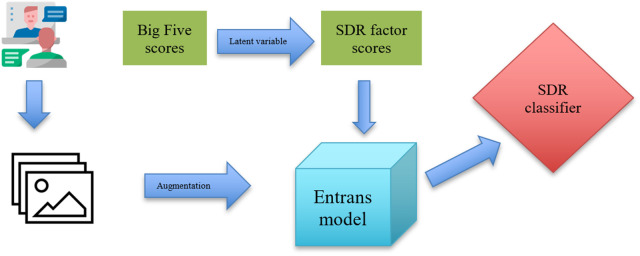
Model training pipeline. *Note.* Sequences of images extracted from each participant’s video are utilized to train an EfficientNet-based transfer learning model, designated *Entrans*. Prior to model fitting, data augmentation techniques are applied to the images. *Entrans* are trained to identify patterns within the subjects’ sequences of images, with the objective of accurately predicting their SDR factor scores. The SDR factor scores are extracted as a latent variable from the subjects’ Big Five scores using structural equation modelling.

To construct a robust transfer learning model, we used EfficientNet [64], the state-of-the-art deep neural network for image recognition, as our base model. EfficientNet is based on the convolutional neural networks (CNN, or ConvNet) baseline model EfficientNet-B0 whose architecture consists of several blocks of layers composed of *mobile inverted bottleneck* (MBConv) [64]. Underpinned by EfficientNet, we trained a SDR classifier that took images as input, then output the prediction of SDR in either continuous or categorical values, depending on the training task (see Fig 1 for the model training pipeline). For abbreviation, we designated our model *Entrans*, which stands for **E**fficient**N**et **Trans**fer-learning. We incorporated the weights of ImageNet [65] into our model to optimize *Entrans*’ performance.

**Fig 2:**
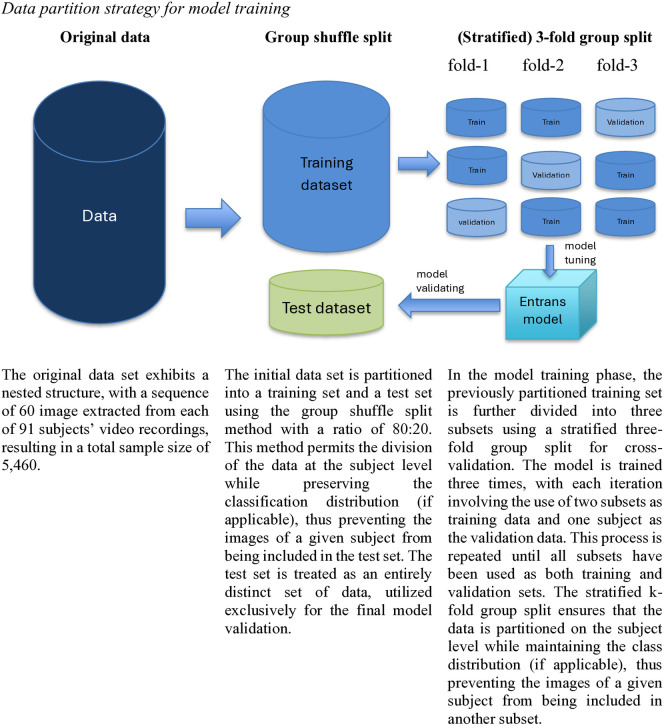
Data partition strategy for model training.

For the purpose of model evaluation, we introduced nested cross-validation in both regression and classification prediction tasks, which consisted of holdout and 3-fold cross-validation (stratification was additionally applied to the classification task). To execute this method, we split the data into training and testing sets (80:20) using *group shuffle split* owing to the nested nature of our input data, which contains a sequence of images for each participant. This method permits the division of the data at the participant level while preserving the data distribution (for the classification of classification), thus preventing the images of a given participant from being included in the test set. This means, the training set contains 72 participants (4320 data points: 72 x 60 images) and the test set contains 19 participants (1140 data points: 19 x 60 images). Subsequently, we trained the model on the training set only, using 3-fold (stratified) cross-validation, which further divided the training set into three subsets, and each subset contains 24 participants (1440 data points: 24 x 60 images). The model was trained with two subsets, and the third subset was used to validate its performance. This process was repeated three times, with each subset being used for both training and validation. It is worth noting that the train and test datasets have distinct participants (thus their sequence of images) to avoid data leakage. The test set, partitioned beforehand, served as unseen data for final performance validation only after *Entrans* was fine-tuned from the training set. To avoid *Entrans* exploiting the image index as a pattern to deceive the prediction, we also remove the participants’ file names to double-blind the model. For further details regarding our cross-validation approach, please refer to Fig 2.

For the model training, we resorted to learning rate scheduler to automatically search for the optimal learning rate. Data augmentation technique applied on the training dataset was randomly shuffled in each training seed, thus differentiating the training sets among the three training rounds. To evaluate model performance for the regression task, we relied on the Root Mean Squared Error (RMSE) to assess the margin of error distancing predicted and actual SDR values, as calculated using the Equation (1). To support the result, we also computed Spearman’s correlation coefficient to illuminate the effect size of the model performance.


$$RMSE\;=\sqrt{\frac{1}{n}\sum {\mathrm{\backslash nolimits}}_{i=1}^{n}{\left(y_{i}-{\hat{y}}_{i}\right)}^{2}}$$ (1)

As for the classification task, considering that our data have imbalanced classes, the choice of the evaluation metrics required that they should reflect the model performance on each of the classes. Popular metrics such as Accuracy, Precision, and Recall are often insufficient as they fail to provide a complete picture of the model’s behavior. Instead, we relied on the Area under the ROC Curve (AUC, see [66]). The AUC is a binary classifier metric used to assess classification performance. It is derived from the *Area under the ROC curve*, which is the integral of the true positive rate (TPR) against the false positive rate (FPR) over the range of thresholds. It can be calculated using Equation (2), where $\int_{0}^{1}$ denotes the range of the limits of integration between 0 and 1; TPR(FPR) represents the true positive rate as a function of the false positive rate and shows how TPR changes with different values of FPR; d(FPR) indicates the differential element of the false positive rate, which is the variable with respect to which the integration is performed. In the present case of SDR classification, the AUC can be interpreted as follows: given a randomly selected pair of individuals where one displays a higher SDR tendency and the other lower SDR tendency, there is a chance that our model will correctly rank the person with higher SDR tendency above the one with lower SDR tendency. In our model training approach, we have also relied on the focal loss function to better capture the deviation between the actual and predicted values, as this function is tailored to handle cases with imbalanced classes [67].


$$\text{AUC}\;=\;\int {\mathrm{\backslash nolimits}}_{0}^{1}TPR(FPR)d(FPR)$$ (2)

In the post hoc phase, our classification prediction operation will allow us to employ the Grad-CAM (Gradient-weighted Class Activation Mapping, see [68])—an Explainable AI method for visually inspecting the prediction decision of large ConvNet-based models—to gain insight into the patterns identified by *Entrans* in discriminating participants’ SDR tendency. Grad-CAM works by examining the final convolutional layer in the neural network model and computing the gradient derived from that layer. The Grad-CAM technique produces a heatmap visualization for a given class label, enabling model trainers to verify the areas in the image that the model is focusing on. This technique is more suitable for classification tasks, providing insights into the parts of an image that are most relevant to the network’s predictions of a given class.

In the next section, we present the results of two analyses in which we trained our transfer learning-based Entrans model to predict continuous SDR scores in the regression prediction task and binary SDR categories in the classification prediction task. We adjusted the *Entrans* model to accommodate the specific needs of the classification task. All the results refer to the prediction on the unseen data (the test dataset) that was held out for final validation after we fine-tuned *Entrans* with 3-fold cross-validation. To provide a more comprehensive evaluation of *Entrans*, we have conducted an iterative process of model training using other commonly deployed yet robust machine and deep learning algorithms, including random forest and simple convolutional neural network (CNN) for the regression task, and CNN-LSTM (long short-term memory) for the classification task. Random forest is an ensemble learning type of algorithm that operates by constructing a vast number of decision trees in situ during the training phase. It is often observed that this approach yields satisfactory results in the context of regression-type prediction (see [69]). The simple CNN that we constructed for comparison comprised two-layer convolutional neural networks (including one CNN layer and one global average pooling layer), which can be considered as the rudimentary version of *Entrans*. The architecture of the CNN-LSTM model is designed to capture both spatial and temporal information of the input data, thereby enabling the CNN layers to extract features and the LSTM layers to support next sequence prediction (see [70]). The online translation tool Google Translate, as well as numerous large language models currently in use, have been influenced by this architecture. CNN-LSTM can be thus considered as a strong competitor to our model.

For further technical details on a subset of our data, the architecture of our and other competing models, our model training pipeline, including Python source code, and the post hoc model evaluation procedure to replicate our experiment, please refer to our Supplementary Document, shared via OSF(https://osf.io/w46jk/?view_only=a632e65e441e42d69dd98c00708eae77).

## Results

### SDR estimation

The robust maximum likelihood estimator yielded an acceptable model fit [5] (*X*^2^ = 4.93, **p* *= .425, CFI = 1, RMSEA < .001, SRMR = .053). The loadings showed negative values for all traits, namely −.01 (Extraversion), −.16 (Openness), −.30 (Emotional Stability), −.37 (Conscientiousness), and −.74 (Agreeableness). To ensure that higher scores reflected greater SDR, we multiplied the extracted SDR factor score by minus 1. Based on the loadings, it can be posited that this factor most likely represents communal impression management style, marked by higher loadings on Agreeableness, Conscientiousness, and Emotional Stability (Paulhus, 2002). This finding is consistent with meta-analytical findings that reveal a strong susceptibility of Conscientiousness and Emotional Stability to SDR [5]. The computed SDR estimate is zero-centered, ranging from −1.60 to 1.66, with a standard deviation of.78. The estimated construct reliability of McDonald’s omega is .60, supporting the assumption of a substantial common variance. The extracted factor score for each respondent was then used as the criterion for SDR prediction.

### SDR prediction

#### Main analysis: Regression prediction task.

As Table 1 illustrates, *Entrans* achieved a root mean square error (RMSE) of .27 and mean squared error (MSE) of .07 when predicting continuous SDR scores on the unseen data. Additionally, the model yielded a correlation coefficient (*ρ*) of .27, which suggests a moderate degree of correlation between the predicted and actual values. As there is no established benchmark for determining the optimal MSE or RMSE value, two additional prominent machine and deep learning models have been trained and included in the comparison: random forest and a simple CNN.

**Table 1:** Regression prediction task.

Model/metrics	*MSE* [95% CI]	*RMSE* [95% CI]	*Ρ* [95% CI]
Random forest	.12[.11,.13]	.34[.33,.36]	−.11[−.17, −.05]
Simple CNN	.07[.06,.07]	.26[.25,.27]	.16[.11,.22]
***Entrans***	**.07**[.07,.08]	**.27**[.27,.29]	**.27**[.16,.28]

*Note*. In the context of root mean square error (RMSE) and mean square error (MSE), lower values indicate greater robustness of the model. Conversely, for Spearman’s correlation coefficient *ρ*, the greater the value, the more robust the model. The 95% confidence interval was calculated using Bootstrapping method, consisting of resampling the data with replacement for 1000 times.

**Fig 3:**
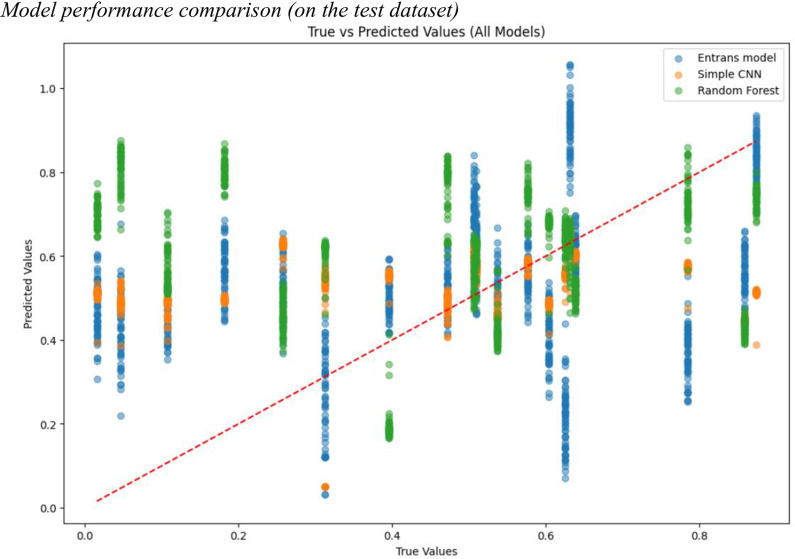
Model performance comparison (on the test dataset).

Table 1 presents the results of the comparison. We observed that the random forest was not as effective as the simple CNN and *Entrans*, with a RMSE of .31 and MSE of .12. It is noteworthy that the simple CNN achieved a remarkably competitive result, with a RMSE of .26 and MSE of .07, which was on par with *Entrans*. The simple CNN exhibited less overfitting in the earlier phase of training than *Entrans*, yet subsequently ﬁnished in an unfavorable local minimum. When the three models were compared in terms of Spearman’s correlation coefficient *ρ*, *Entrans* outperformed both simple CNN (*ρ *= .16) and random forest (*ρ *= −.11). The 95% confidence interval provided in Table 1 showed a head-to-head level between the simple CNN and the *Entrans* model in terms of prediction error rate; however, *Entrans* achieved a significantly higher confidence interval [.16,.28] than the simple CNN [.11,.22] in terms of correlation. This trend is corroborated by the visualization of the prediction versus actual scores, as illustrated in Fig 3. This figure provides insight into the prediction behaviors of the benchmarked models: our *Entrans* model tended to predict either correctly or closer to the true labels across the entire score range, while the simple CNN seemed to adopt a more conservative approach, predicting closer to the true labels but with less accuracy. This difference may explain why *Entrans* achieved a higher Spearman correlation coefficient.

#### Supplementary analysis: Classification prediction task.

As before, we trained the *Entrans* model for classification prediction using 3-fold cross-validation on the training dataset. Subsequently, the prediction performance of the fine-tuned model on the unseen test dataset achieved an AUC score of .71 (see Table 2), suggesting that it demonstrated an above-average ability to distinguish between a person’s lower and higher SDR tendency compared to a random guess. To illustrate this point, a random guess rate was calculated on our dataset, which exhibited a skewed class distribution. Consequently, the chance of guessing correctly the lower SDR was 56.25%, whereas the chance of guessing correctly the higher SDR became challenging, with a probability of just 6.25%, as specified in Table 2. Table 2 also demonstrated that *Entrans* achieved *F*_*1*_ scores of .66 for the case of lower SDR and .33 for the case of higher SDR. Table 3 depicted the confusion matrix issued from the unseen data (i.e., the test dataset). It is important to clarify that the instances refer to one or more images derived from the sequences of images of participants, rather than the participants themselves. It should be interpreted that the model’s predictions regarding higher or lower SDR are contingent upon the activation of particular behaviors (such as smiling) by the participant as represented in the corresponding image (see also the Grad-CAM explanation below). The resulting sensitivity was 68.89%, the specificity 52.60%, the PPV (positive predictive value) 21.42%, and the NPV (negative predictive value) 90.02%.

**Table 2:** Classification prediction task.

Model/metrics	*F*_*1*_ *-* ${SDR}_{lower}$	*F*_*1*_ *-* ${SDR}_{higher}$	*AUC* [95% CI]
Random guessing	.56	.06	
CNN-LSTM	.75	0	.5[.47,.53]
***Entrans***	**.66**	**.33**	**.71**[.65,.74]

*Note*. The random guessing was calculated based on the class distribution. It should be noted that 75% of the data represents the proportion of the ${SDR}_{lower}$ cases. Consequently, the likelihood of correctly guessing low SDR is.75*.75, while the chance of correctly guessing the ${SDR}_{higher}$ is.25*.25.

**Table 3:** Confusion matrix (on the Test dataset).

		Predicted	
		${SDR}_{lower}$	${SDR}_{higher}$
**Actual**	${SDR}_{lower}$	505	455
	${SDR}_{higher}$	56	124

*Note*. The confusion matrix delineates the instances that were accurately (in grey shade) and inaccurately predicted within the test dataset. The resulting sensitivity was 68.89%, the specificity 52.60%, the PPV (positive predictive value) 21.42%, and the NPV (negative predictive value) 90.02%.

**Fig 4:**
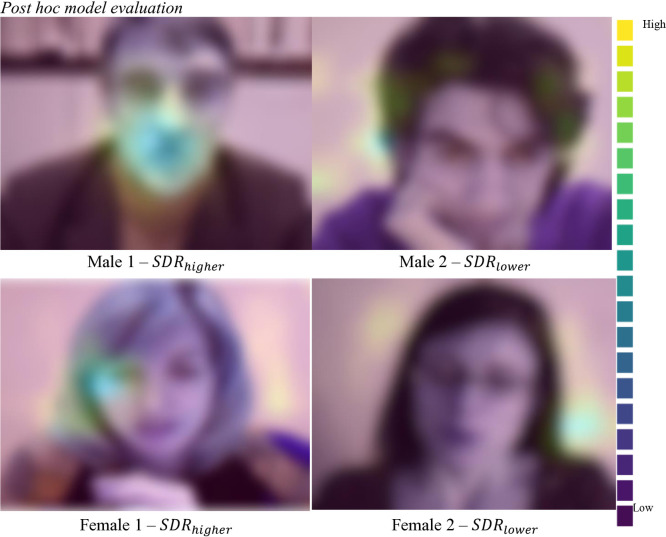
Post hoc model evaluation. *Note*. The heat maps generated by Grad-CAM are superimposed on the actual images of the sample participants. The color map used is based on the *viridis* type. In the four images above, as reference to the color bar in the right side, the brighter colors (yellow, green, and blue) indicate the regions upon which *Entrans* relied to make prediction. To ensure participant privacy, we blurred all the images.

To further investigate the resilience of *Entrans*, we administered the CNN-LSTM model to present a more competitive benchmark. In our test, despite the sophistication of its design, the CNN-LSTM exhibited poor performance in distinguishing between lower and higher, as evidenced in Table 2. In fact, the model adopted a strategy similar to that of the zero-rule model, which employed a simple approach to predict the majority class. This resulted in a *F*_*1*_ score of .75 for lower SDR and 0 for higher SDR, with an AUC score of .5, which was equivalent to guessing. A post hoc evaluation was conducted on the classification task using Grad-CAM in order to gain a deeper understanding of the patterns that *Entrans* leveraged in order to determine the lower or higher SDR. The results of this evaluation are presented in Fig 4. By visualizing the areas identified as markers by *Entrans* within the image sequences, it can be seen that the model prioritized the regions around the mouth, cheeks, and eyes as the most important indicators of higher SDR; conversely, the lack of obvious features when classifying lower SDR participants seems to be the best description of a classification rule. This supplementary evidence further corroborates our previous findings from the regression in that *Entrans* had learnt the ability to detect the presence of SDR patterns/features.

## Discussion

The present study presents a proof-of-concept heralding the utility of the deep learning approach to advance our knowledge regarding the association of SDR exhibited in questionnaires and in self-presentation. Our study shows that a sophisticated deep learning model might be able to identify nonverbal cues obtained from an interview-like situation as indicators for the presence of SDR in a questionnaire. Using transfer learning, we experimented with a model and examined how well it was able to detect participants’ tendency to use SDR tactics when answering personality assessment questionnaires. Our results show that our model *Entrans* performs fairly in predicting such a tendency on the basis of participants’ video presentation.

We leveraged GradCAM to interpret the patterns (regions of interest) identified by our model in a holistic manner and discerned that the middle and lower part of the face could be a key area to detect the occurrence of SDR. A reasonable explanation would be that the middle and lower portions of the face correspond to several nonverbal cues, such as chin movement, mouth opening, eyebrow elevation, and so on, which may be related to nodding and smiling, as well as other forms of facial expression. It could be that nonverbal behaviors – exemplified by these facial expressions – reflect an individual’s impression management techniques. The outcome echoes the hypothesis positing that people consciously regulate their nonverbal behaviors to present themselves in a way they find desirable [56]. Moreover, nonverbal behaviors such as smiling have been identified as impression management techniques, used in job interviews [71]. The finding is also in line with past findings that have supported the association between impression management and facial expressions such as smiling [72], eye contacts, and nodding [73]. Importantly, lower SDR was not signified by such tell-tale features but rather by the absence of indicative features. This could reflect the notion that SDR is a continuous construct (e.g., [43]), and higher levels manifest in more pronounced behaviors, not qualitatively different behaviors. It has to be stressed here that the connection between potential impression management in the self-presentations and SDR in a questionnaire provides support to the notion that impression management tactics applied in different assessment situations might be derived from a common source. This implies that there may be systematic relations between different forms of SDR behavior, pointing to an overarching SDR variable whose effects may spread over different assessment domains. Our findings echo those of Ingold and her colleagues [74], who found that job candidates who use impression management tactics during an interview tend to engage in biased responses on personality questionnaires. To the extent that facial behavior patterns in self-presentation emerge as important indicators of SDR in our experiment, we see potential for using short video self-presentations to develop deep learning-based toolkits. These toolkits could alert recruiters when a candidate is engaging in impression management tactics.

### Linking SDR across assessment methods

Previous research has investigated SDR in questionnaires or in interviews, but the relation between SDR in these two assessment measures has not been thoroughly explored. This study presents findings which suggest such a relation, linking SDR in questionnaires and interviews. Our results demonstrate that the SDR behavior exhibited by participants in their video self-presentations is indicative of that observed in their questionnaire responses. This indicates a common source for participants’ SDR behavior in both questionnaire and interview-based assessments.

Previous research suggests that SDR behavior occurs across all assessed personality traits within a single questionnaire, suggesting that SDR may reflect a unidimensional construct [9,13,43,75,76]. Similarly, research on interviews indicates that the ability to identify criteria is associated with SDR behavior (impression management or faking) across multiple assessed dimensions [6]. As such, the idea that SDR behavior in each assessment method may be related to a unidimensional construct has found empirical support for both questionnaires and interviews. We extend this body of literature by theorizing that there are systematic relations in the manifestations of these constructs, albeit obvious differences between questionnaire distortion and self-presentation distortion. With respect to questionnaires, process models of faking behavior focus on assessing the importance of each item to the SDR goal as well as the actual distortion of the response scale [77,78]. With respect to interviews, or overt behavior in general, the empirical evidence primarily focuses on speech [10] rather than on specific nonverbal behavior. By contrast, our deep learning model detects SDR by inferring from nonverbal cues. Despite concerns that nonverbal indicators of SDR may reflect alternative constructs—such as fidgeting potentially signaling anxiety rather than distortion—we nonetheless illustrate the potential for future research on SDR to explore a specific category of nonverbal cues and their evolution over time by establishing a systematic relationship between SDR behaviors identified in a questionnaire and those exhibited in self-presentation scenarios.

### Practical implications

First of all, it has to be noted that the current proof-of-concept does not reach levels of accuracy, warranting actual practical implications. Instead, we want to showcase this approach, thereby hoping to spark more research with larger samples. Nevertheless, the current works shows that using deep learning approaches to detect SDR has potential in the context of personnel selection. While interviews and personality questionnaires are popular instruments for evaluating job applicants’ work performance, studies on SDR (e.g., [79,80]) agree that the effectiveness of these assessments can be compromised if applicants engage in SDR tactics. Personnel selection results may be impaired if vendors of personality assessments or hiring managers do not adequately address the challenges posed by SDR. Traditional methods used to control SDR in the personnel selection process are common, but they can be time-consuming, costly, even untrustworthy (e.g., the validity of lie scales is still highly contested). A deep learning-based SDR detection system, as proposed in the present study, might eventually provide an opportunity for organizations of any size to automate bias detection during the selection process.

Where appropriate, future research in this domain should further integrate Explainable AI methodologies to analyze the decision-making patterns of deep learning algorithms and identify specific behaviors associated with SDR in a more comprehensive fashion than we did in this proof of concept. This approach will empower recruiters to recognize such behaviors more effectively. For instance, our experiment suggests that certain facial expressions may be indicative of the application of SDR tactics. By tracking the frequency with which candidates display these behaviors during interviews, recruiters could determine whether further assessment is warranted to enhance the accuracy of their evaluations. To support such conclusions, future studies should investigate the reasoning mechanisms underlying deep learning models, as demonstrated in our research. Moreover, such approaches can help to better understand the psychological processes behind the latent construct of SDR.

### Limitations

Despite its potential, the present study has several limitations. First, *Entrans* was trained on a relatively small sample size. Although we have implemented various strategies to mitigate this issue with regard to model overfitting, the concerns on the generalizability of our results may still remain. Our current model structure may still be too complex for the limited data size. We therefore recommend that future research experiment with a larger sample size to enhance the generalizability of the findings. The optimal sample size is contingent upon the specific tasks being addressed, and there is no definitive answer to this question. Additionally, our model relied on image sequences as input data to identify visual patterns for detecting SDR. It is important to acknowledge that other nonverbal cues, such as vocal characteristics, may also serve as significant indicators of SDR [81]. Consequently, the development of a multimodal transfer learning model could represent a promising direction for improving *Entrans*’s performance. Furthermore, we advise that future studies prioritize the collection of balanced and diverse data, encompassing factors such as gender, age, ethnicity, minority status, and social background. This approach will help ensure that the training data does not adversely affect the generalizability of the deep learning model.

In this preliminary study, we made the decision to simplify certain operational procedures related to the model. For instance, we employed a binary value (lower and higher SDR) as our predicted dependent variable, based on a 75/25 cutoff threshold. While this approach is supported by previous research, we acknowledge that it may not fully capture the complexity of the phenomena under investigation, and we encourage future researchers to consider more sophisticated research designs. In addition, although we experimented a classification model as a means of corroborating our initial findings, we recognize that this choice may have limitations and may not provide a comprehensive understanding of SDR. We emphasize that our primary goal is to develop a regression solution that allows the model to predict continuous SDR scores without artificial transformations. While the classification task has its merits, we urge caution in interpreting the results derived from it, as they should be viewed as preliminary and not definitive. We assert that this simplification serves as a foundational step in our exploration of SDR, but further investigation is necessary to validate our findings.

In our research design, we relied on well-documented evidence [4,18,20,77] to construct a latent variable model that extracts SDR factor scores from the Big Five personality scores of the sample participants. While this approach provides a practical solution for developing an SDR detection system without the complexities associated with experimental methods or other more intricate SDR assessments—many of which also face challenges related to validity—we acknowledge that some may question whether our model is truly predicting a SDR factor rather than a Big Five trait. To address this concern, we employ the Grad-CAM technique to elucidate the decision-making process of the *Entrans* model. The results demonstrate that our model identifies nonverbal patterns linked to facial regions associated with smiling behavior, particularly in the middle and lower parts of the face. This finding aligns with previous research suggesting that job applicants often employ nonverbal behaviors, such as smiling, as impression management strategies during interviews [71,82] and further suggests that the model is predicting SDR-related behaviors rather than merely reflecting Big Five traits. However, we urge caution in interpreting these results, as the relation between nonverbal cues and SDR is complex and may be influenced by various contextual factors. Further research is necessary to validate and substantially extend these findings and ensure that our model’s predictions are robust across different settings.

That said, we recognize that the theoretical construct of SDR is complex. It is essential to highlight that our study does not distinguish between the conscious (i.e., impression management) and subconscious (i.e., self-deceptive enhancement, as outlined by [4,83]) SDR behaviors of the sample participants. Future research may benefit from employing more nuanced operationalizations of SDR to train deep learning models capable of detecting various types of SDR tactics. One potential limitation of our study is that the data were collected in a laboratory setting rather than from actual applicants. Laboratory environments have been criticized for generating larger SDR effects than those typically observed in real-world contexts [5]. If our data exhibited larger SDR effects, the models might present an unrealistically strong signal, which could result in an overestimation of their predictive capabilities. However, it should be noted that participants in our sample were not instructed to deceive but were only asked to simulate an interview scenario. Nevertheless, it would be valuable for future research to validate our proposed approach using data from real applicants.

Our initial efforts to interpret a complex deep learning model with Grad-CAM technique have produced interesting insights; however, we recognize the need for more efficient tools specifically designed for neural network architectures that can provide deeper insights into their predictive decisions. Unlike traditional machine learning algorithms, the architecture of very deep neural networks, such as EfficientNet implemented in our research, is often too intricate to allow for fully satisfactory model interpretation. Nevertheless, we believe that interpreting algorithmic decisions is essential for uncovering previously hidden patterns and refining existing theories. This process is also critical for addressing potential biases in personnel selection algorithms, particularly those related to race, gender, or skin tone (see [84]). In this study, we employed one sophisticated Explainable AI technique to facilitate the interpretation of our model’s decisions. We contend that there is a pressing need for a more robust, informative, and user-friendly method tailored specifically for regression prediction tasks within the realm of computer vision. Such advancements would significantly enhance our ability to reliably uncover the decision-making processes and patterns identified by complex neural network models.

## Conclusion

Minimizing the impact of SDR during the hiring process is a critical responsibility for any organization aiming to recruit employees without falling prey to information distortion. While there is an increasing number of commercial vendors promoting their “AI” talent assessment tools, it is important to note that none have effectively addressed the challenge of SDR. In this paper, we present a proof-of-concept that illustrates how organizations can leverage ML-related approach to detect job applicants’ SDR tendencies. Our study highlights the potential of integrating deep learning methods with psychological theory, not only to develop highly predictive models but also to advance theoretical understanding.

Our findings indicate that SDR, which manifests in self-presentations and questionnaires, may stem from a common source. Additionally, we identified significant nonverbal cues and formulated hypotheses regarding the specific nonverbal behaviors associated with SDR. We also connected these behaviors to existing research on the dynamics of liking and impression management in job interviews. We hope that our work will inspire additional research into uncovering the relationships between abstract traits and their behavioral manifestations.

## Supporting information

S1 File Transparency and openness. (DOCX)
