# Supplementary material for: Leveraging deep learning for the detection of socially desirable tendencies in personnel selection: A proof-of-concept
Source: PLoS One. 2025 Aug 5;20(8):e0329205. doi: 10.1371/journal.pone.0329205 (PMC12324085; doi:10.1371/journal.pone.0329205)
Supplement: S1 File — (DOCX) [file pone.0329205.s001.docx]

# TRANSPARENCY AND OPENNESS

We report how we determined our sample size, all data exclusions (if any), all data inclusion/exclusion criteria, whether inclusion/exclusion criteria were established prior to data analysis, all measures in the study, and all analyses including all tested models. If we use inferential tests, we report exact p values, effect sizes, and 95% confidence or credible intervals.

The information needed to reproduce all of the reported results is not openly accessible due to data privacy; this applies to sample participants’ video data and their corresponding biometric features. A subset of the data that does not reveal participants’ identity (i.e., their extracted SDR scores) is available via our shared OSF repository.

We confirm that there is sufficient information for an independent researcher to reproduce all of the reported methodology. All studies presented throughout this article were conducted according to standard ethical guidelines.

This study was not preregistered.

We confirm that all the scripts, code, and outputs needed to reproduce the results are provided via the shared OSF repository as below:

https://osf.io/w46jk/?view_only=a632e65e441e42d69dd98c00708eae77
